# Factors affecting the timing of adiposity rebound

**DOI:** 10.1186/1687-9856-2013-S1-O47

**Published:** 2013-10-03

**Authors:** Satomi Koyama, Katsura Kariya, Go Ichikawa, Naoto Shimura, Toshimi Sairenchi, Osamu Arisaka

**Affiliations:** 1 Department of Pediatrics, Dokkyo Medical University, Japan; 2 Department of Public Health, Dokkyo Medical University, Japan

## Background

The age of adiposity rebound (AR), when body mass index (BMI) starts to rise after infancy, is thought to be an origin of obesity in later life. We have already reported that children who exhibited an earlier AR were associated with the higher BMI value and atherogenic metabolic status at 12 years of age. We investigated which factors influenced on an earlier AR, birth weight, initial feeding, family history, meals or exercise.

## Methods

A total of 533 children in the community were enrolled in the study. Serial measurements of BMI from 4 months to 12 years were carried out prospectively. We calculated the age of AR, defined as the age which the lowest BMI occurred during this period. The subjects were divided into 2 groups according to BMI at 3 years is bigger than at 1.5 years (earlier AR group) or not (later AR group). We asked the answering to the question sheet about weight at birth, initial feeding (breast-feeding, bottle-feeding or mixed feeding), family history, meals, and exercises of their parents when children were at 3 years old. We also analyzed which BMI predicted the obesity at 12 years old, 4, 8, 12, 18 month or 2, 3, 4, 5 or 6 years by using ROC analysis.

## Results

Weight at birth was associated with earlier AR if birth weight was over 3500g, but was not associated with the timing of AR if it was between 1500g and 3000g. Initial feeding was not related to the timing of AR and the frequency of obesity at 2 years old. None of the breast-feeding subjects showed severe obesity at 12 years old. The factors as follows were associated with later AR; eating breakfast every day, not eating snacks, non-obese father, the first baby, going to kindergarten. Contrary to expectation the habits of drinking sweet beverages and playing outside were not related to earlier AR. BMI at over 2 years old predicted to the obesity at 12 years old, but BMI in the infantile periods did not.

## Conclusion

This study showed that obesity at 12 years old was associated with weight gain over 2 years old, but not with the weight gain during infantile period.

